# After Intracerebral Hemorrhage, Oligodendrocyte Precursors Proliferate and Differentiate Inside White-Matter Tracts in the Rat Striatum

**DOI:** 10.1007/s12975-015-0445-3

**Published:** 2016-01-08

**Authors:** Michael J. E. Joseph, Jayalakshmi Caliaperumal, Lyanne C. Schlichter

**Affiliations:** Krembil Research Institute, University Health Network, Krembil Discovery Tower, Room 7KD-417, 60 Leonard Street, Toronto, ON M5T 2S8 Canada; Department of Physiology, University of Toronto, Toronto, ON Canada

**Keywords:** Hemorrhagic stroke, Myelinated axon tracts, Corticostriatal axons, Peri-hematoma recovery, Oligodendrocyte maturation, Post-stroke recovery

## Abstract

Damage to myelinated axons contributes to neurological deficits after acute CNS injury, including ischemic and hemorrhagic stroke. Potential treatments to promote re-myelination will require fully differentiated oligodendrocytes, but almost nothing is known about their fate following intracerebral hemorrhage (ICH). Using a rat model of ICH in the striatum, we quantified survival, proliferation, and differentiation of oligodendrocyte precursor cells (OPCs) (at 1, 3, 7, 14, and 28 days) in the peri-hematoma region, surrounding striatum, and contralateral striatum. In the peri-hematoma, the density of Olig2^+^ cells increased dramatically over the first 7 days, and this coincided with disorganization and fragmentation of myelinated axon bundles. Very little proliferation (Ki67^+^) of Olig2^+^ cells was seen in the anterior subventricular zone from 1 to 28 days. However, by 3 days, many were proliferating in the peri-hematoma region, suggesting that local proliferation expands their population. By 14 days, the density of Olig2^+^ cells declined in the peri-hematoma region, and, by 28 days, it reached the low level seen in the contralateral striatum. At these later times, many surviving axons were aligned into white-matter bundles, which appeared less swollen or fragmented. Oligodendrocyte cell maturation was prevalent over the 28-day period. Densities of immature OPCs (NG2^+^Olig2^+^) and mature (CC-1^+^Olig2^+^) oligodendrocytes in the peri-hematoma increased dramatically over the first week. Regardless of the maturation state, they increased preferentially inside the white-matter bundles. These results provide evidence that endogenous oligodendrocyte precursors proliferate and differentiate in the peri-hematoma region and have the potential to re-myelinate axon tracts after hemorrhagic stroke.

## Introduction

Intracerebral hemorrhage (ICH) comprises 10–20 % of all stroke cases [1] and is a result of spontaneous rupture of brain arterioles, which produces a hematoma in the brain parenchyma. The overall mortality rate is ∼40 % by 1 month [2], >70 % at 5 years [3], and few survivors regain functional independence [4]. With no drug therapies in clinical use, current treatment focuses on supportive care and reducing the impact of the damage [5, 6]. The primary injury phase occurs within minutes to hours after the onset of bleeding, and during this time, the hematoma causes local tissue destruction [7, 8]. Then, cellular debris and breakdown of blood components initiate a secondary injury phase, which can last for days to weeks in the peri-hematoma region adjacent to the hematoma [7, 8].

Following ICH, brain cells of all types die inside the hematoma, which forms a non-vascularized blood clot. Therapeutic approaches to treating ICH therefore focus on the peri-hematoma region surrounding the lesion, and on preventing damage from spreading. In the peri-hematoma, in addition to neuronal injury, white matter is damaged but potentially rescuable. In humans, white-matter damage was reported in 77 % of ICH patients [9]. Most preclinical, animal studies of ICH have focused on gray matter [10, 11], with very little research on white-matter damage and its progression. CNS white matter is comprised of axons, myelin sheaths that enwrap them, and oligodendrocytes, the cells that produce myelin [12]. Myelinated axons are essential for CNS communication, with millions of afferent and efferent axons arranged in white-matter tracts. Because of its crucial role, even small white-matter lesions can produce devastating neurological deficits [13]. Functional recovery and efficacy of potential treatments will require both axon survival and re-myelination.

Hypertensive ICH often occurs in the human striatum [14]. We have been exploiting the well-established collagenase model of ICH in the rat striatum and previously found that progressive, extensive damage to myelin bundles and axons during the first week correlated temporally and spatially with inflammation and microglial activation [15–18]. However, the fate of axons and oligodendrocytes is not known. In order to identify therapeutic targets for rescuing white matter after ICH, a deeper understanding of the cellular pathology is needed. The paucity of information raises several important questions. Do oligodendrocyte-lineage cells remain in the damaged region after ICH? If so, do they proliferate and remain as non-myelinating oligodendrocyte precursor cells (OPCs), or do they differentiate into mature cells? Does oligodendrogenesis occur within the peri-hematoma? Here, we addressed these questions from 1 to 28 days after collagenase-induced ICH in the rat striatum.

## Materials and Methods

### Animals and Surgery

Male Sprague-Dawley rats weighing 300–350 g (Charles River, Saint-Constant, Quebec, Canada) were randomly assigned to either the saline control (*n* = 3) or ICH group (*n* = 3 or 4 per time point). They were housed at two rats per cage in a temperature- and humidity-controlled room under a diurnal light cycle (lights on from 6 am to 6 pm), and provided with food and water ad libitum.

Surgical procedures were performed aseptically. Rats were anesthetized with isoflurane (4 % induction, 2 % maintenance), placed in a stereotaxic frame (David Kopf Instruments, Tujungam, CA), and a 1-mm-diameter burr hole was drilled into the skull 3.0 mm lateral and 0.1 mm anterior to bregma. A 26-gauge needle attached to a micro-pump (model UltraMicroPump II; World Precision Instruments, Sarasota, FL) was inserted 6.0 mm deep from the surface of the skull. Rats were injected with 0.5 μL of sterile 0.9 % saline (controls) or saline containing 0.03 U of type IV collagenase (Sigma-Aldrich, Oakville, Ontario, Canada) for experimental ICH. The injection rate was 100 nL/min. The dose of collagenase was chosen after a titration pilot study so that the hematoma was restricted to the striatum, with sufficient tissue remaining to have regions of both peri-hematoma and surrounding striatum. The wound was closed with suture clips, and Xylocaine was delivered subcutaneously near the injection site to reduce post-operative pain. Body temperature was maintained throughout the surgery and recovery period using a thermostatic heating pad. All animals regained consciousness within 10 min, and only rats with an ICH demonstrated an ipsilateral turning bias. Their ability to eat, drink, and groom themselves was not impaired, and no rats died because of surgery or ICH induction.

### Tissue Preparation

At 1, 3, 7, 14, or 28 days after surgery, rats were euthanized by an overdose of isoflurane. They were perfused through the heart with 150 mL of phosphate-buffered saline (PBS), followed by 150 mL of 4 % paraformaldehyde (pH 7.4). Brains were removed, post-fixed in 4 % paraformaldehyde overnight, and then placed in 10 % sucrose for 24 h followed by 30 % sucrose for 48 h. The brains were cut coronally or sagittally and embedded in tissue-freezing medium (Ted Pella, Redding, CA). Then, 16-μm-thick sections were cut using a cryostat (model CM350S; Leica, Richmond Hill, Ontario, Canada), mounted on glass slides, and stored at −40 °C until used.

### Cresyl Violet and Immunohistochemistry: Staining and Analysis

Hematoma development at each time point was examined in coronal and sagittal sections using cresyl violet. Frozen brain sections were initially dehydrated in ethanol to remove phospholipids and fixation chemicals [19] and then rehydrated in distilled water for 1 min before adding 0.25 % cresyl violet (Sigma-Aldrich) in a 200 mM acetate buffer (pH 4.0) until well stained [20]. The sections were immersed for 1 min in 0.25 % glacial acetic acid in ethanol to differentiate the white matter, incubated for 4 min in CitriSolv (Fisher Scientific, Pittsburgh, PA) to clear the unstained tissue, and then mounted using DPX (Sigma-Aldrich). Images were made using a flatbed scanner (model HP Scanjet 4070; Hewlett-Packard, Palo Alto, CA).

Immunohistochemistry was used to examine the extent of white matter injury and the density, proliferation, and differentiation of oligodendrocyte-lineage cells. Frozen brain sections were thawed at room temperature and rehydrated for 20 min in a buffer containing 1 M PBS at pH 7.5 (Wisent Bioproducts, St-Bruno, Quebec, Canada), 0.1 % bovine serum albumin (Sigma-Aldrich), and 0.2 % Triton X-100 (Sigma-Aldrich) (PBT). To reduce nonspecific antibody binding, sections were blocked for 2 h in PBT containing 10 % donkey serum (Jackson ImmunoResearch, Baltimore, PA). For staining, sections were incubated overnight at room temperature in PBT buffer containing 5 % donkey serum and two or three of the following primary antibodies.

To assess myelin and white-matter tracts, we used polyclonal antibodies directed against myelin basic protein (MBP), i.e., rabbit anti-MBP (1:1000; ab40390, Abcam, Toronto, Ontario, Canada) or chicken anti-MBP (1:400; ab106583, Abcam). Axons were labeled with a mouse monoclonal antibody against pan-axonal neurofilaments (pan-NF; 1:1000; 837901, Covance, Montreal, Quebec, Canada). To label all oligodendrocyte-lineage cells, we used antibodies against “oligodendrocyte lineage transcription factor 2” (Olig2), either rabbit polyclonal anti-Olig2 (1:500; AB9610, Millipore, Etobicoke, Ontario, Canada) or mouse monoclonal anti-Olig2 (1:500; MABN50, Millipore). OPCs were identified by double staining with anti-Olig2 and a mouse monoclonal antibody against neural/glial antigen 2 (NG2) chondroitin sulfate proteoglycan (1:500; MAB5384, Millipore). In the adjacent serial brain section, mature oligodendrocytes were identified by double staining with anti-Olig2 and a mouse monoclonal antibody against *adenomatous polyposis coli* (APC; also called CC-1; 1:500; OP80, Millipore). Cell proliferation was monitored with a mouse antibody against Ki67 (1:100; ab15580, Abcam). After labeling with primary antibodies, brain sections were washed in PBT (3 × 10 min) and incubated with appropriate secondary antibodies for 2 h at room temperature in the dark. Secondary antibodies (all from Jackson ImmunoResearch) were applied at 1:200 dilution (Alexa Fluor 488-conjugated donkey anti-mouse, Alexa Fluor 594-conjugated donkey anti-rabbit, Dylight 405-conjugated donkey anti-chicken) and were diluted in PBT and 5 % donkey serum. To label cell nuclei, 4′-6-diamidino-2-phenylindole (DAPI; 1:5000; Sigma-Aldrich) was applied for 10 min. After washing in PBT (3 × 10 min), slides were cover-slipped using Dako Fluorescent Mounting Medium (Dako North America, Inc., Carpinteria, CA) and stored in the dark at 4 °C. To minimize variability, immunohistochemistry for a given antibody combination was conducted on the same day for all rats and time points using the same solutions.

**Fig. 1:**
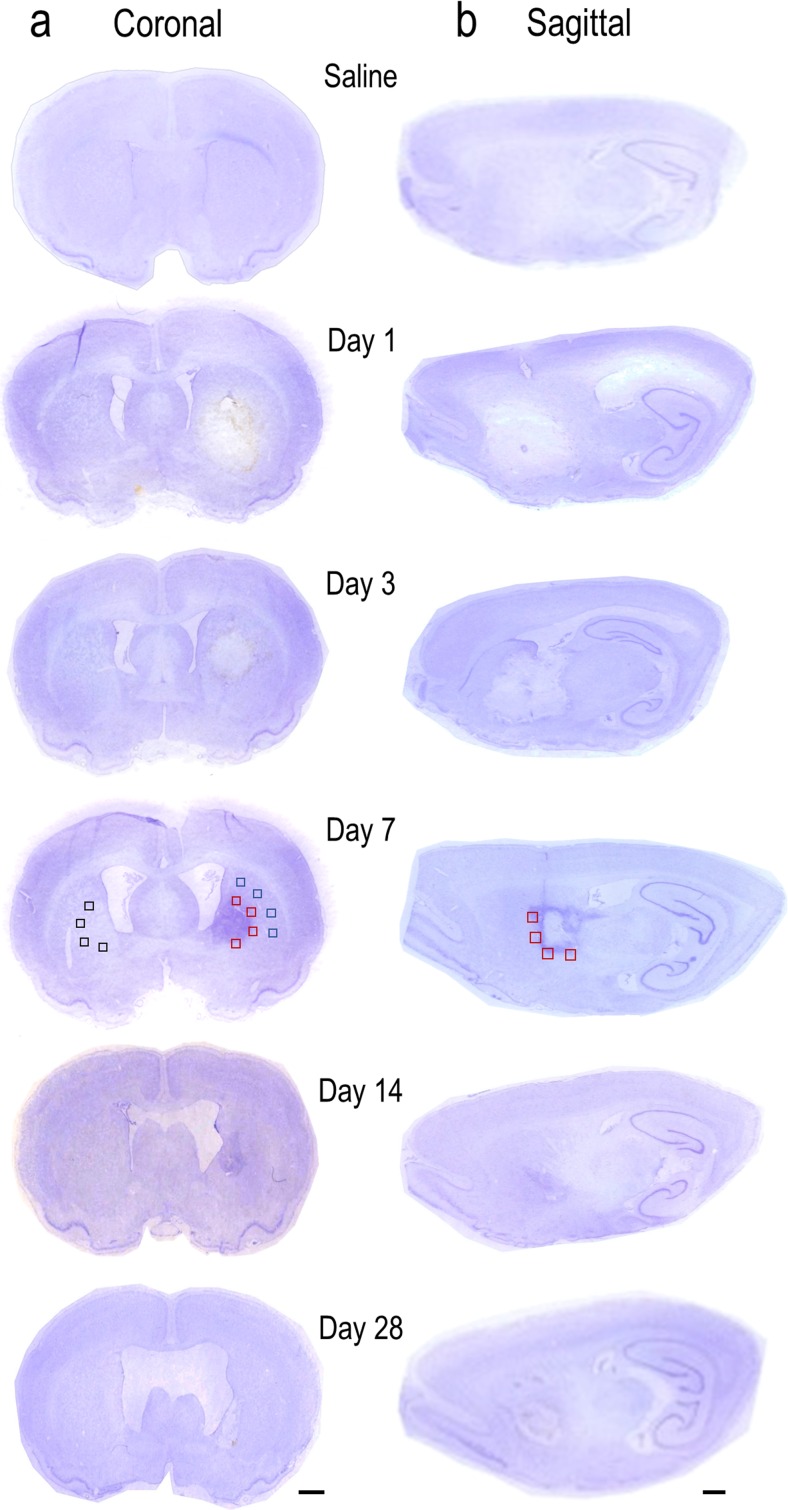
Hematoma development in the first month after ICH. Representative cresyl violet-stained sections from rats on day 1 after saline injection (control) and on days 1, 3, 7, 14, and 28 after injection of type IV collagenase into the right striatum. For day 7 after ICH, *sample boxes* are shown for the peri-hematoma (*red*), surrounding ipsilateral striatum (*blue*), and contralateral striatum (*black*). **a** Coronal sections. The hematoma appears pale, which is especially clear on the right side of the sections on days 1 and 3. On days 7 and 14, the hematoma is smaller and darkly stained. **b** Sagittal sections. *Scale bars* = 1 mm

Antibody-stained sections were examined using a confocal microscope (model LSM700; Zeiss, Oberkochen, Germany) and quantified using ImageJ software, version 1.48v (National Institutes of Health, Bethesda, MD). Images used within each figure were captured with the same pinhole size and detector sensitivity settings, and with short exposure times to avoid fading. Staining areas and cell counts were determined in one coronal or sagittal section per animal, with four sample boxes for each region examined (see schematic in Fig. 1). The hematoma boundary was readily identified by the presence of blood. All peri-hematoma sample boxes were placed immediately adjacent to the hematoma, with one side of each box aligned with the hematoma boundary. When sampling the surrounding ipsilateral striatum, each box was located beyond the peri-hematoma boxes, ventral to the corpus callosum. For the subventricular zone (SVZ), boxes were selected immediately adjacent to the lateral ventricle starting in the dorsal-most region and moving ventrally. For the contralateral region, boxes were randomly placed in the center of the striatum. When cells were counted, all sample boxes were 320 × 320 μm for coronal sections. The boxes were slightly larger (400 × 400 μm) for sagittal sections because of variations in white-matter tract diameter and density, and to have more tracts per image.

### Data Analysis

Oligodendrocyte-lineage cells were quantified at 1, 3, 7, 14, and 28 days after ICH. Cells were counted manually using ImageJ software by an observer who was blinded to the time point and location (i.e., peri-hematoma, surrounding striatum, contralateral striatum). For coronal sections, counts from four sampling boxes at each location and time were averaged, and the mean ± SEM was calculated from three rats at each time point. To assess time-dependent changes within each region, a one-way analysis of variance (ANOVA) was followed by Tukey’s multiple-comparisons test. For sagittal sections, we first quantified the number of cells inside versus outside white-matter tracts in the peri-hematoma region. Then, the area represented by white-matter tracts was calculated by summing the areas of each tract, outlined using the Polygon selection tool on ImageJ. The area outside the bundles was calculated by subtracting the area of the white-matter tracts from the total image area. Counts for oligodendrocyte-lineage cells were converted to densities (number of cells/area). Values are reported as mean ± SEM, from three or four rats at each time point, and comparisons between inside versus outside white-matter tracts were made with a two-way ANOVA followed by Tukey’s multiple-comparisons test. Throughout, differences were considered statistically significant if *p* < 0.05.

## Results

### Myelin Is Damaged but Some Myelinated Axons Survive in the Peri-hematoma After ICH

As expected, there was no hemorrhage in control, saline-injected rats, while a hemorrhage developed when collagenase was injected into the striatum (Fig. 1). At 1 day, collagenase injection produced a large hematoma that was confined to the striatum, and in both coronal (Fig. 1a) and sagittal (Fig. 1b) sections, cell damage was apparent, as shown by reduced cresyl violet staining of Nissl bodies. While a hematoma was evident throughout the 28-day period, the area of diminished cresyl violet staining decreased with time and the blood clot was gradually resorbed. In addition, the lateral ventricles expanded (ventriculomegaly), which is a common outcome after ICH and is due to impaired drainage of cerebrospinal fluid (CSF) [21] or tissue loss [22]. Others, using the same ICH model, have observed similar changes to the size of the hematoma and ventricles for 28 days and longer [22, 23]. The darker staining at 7 and 14 days is due to chromogenic iron that is released from ruptured erythrocytes [24, 25]. In order to quantify changes in the spatial distribution of oligodendrocyte-lineage cells, we used coronal sections and examined three regions: the peri-hematoma, surrounding striatum, and contralateral striatum. Approximate sample locations are illustrated by the boxes in Fig. 1. Then, sagittal sections were used to quantify cells inside versus outside white-matter bundles in the peri-hematoma region. The sample boxes were slightly larger to visualize more bundles and a larger proportion of each bundle.

**Fig. 2:**
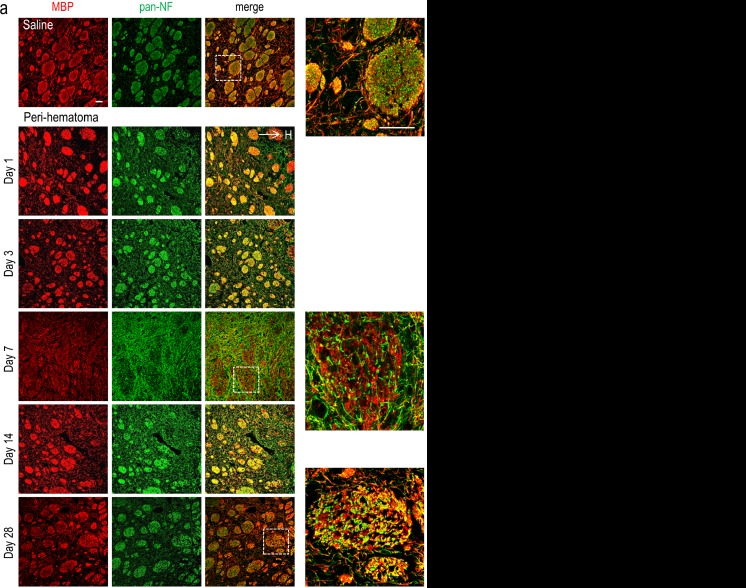
Myelin damage and axon survival in the peri-hematoma after ICH. Representative images of sections double-labeled with an antibody against neurofilaments (*pan*-*NF*; *green*) and an antibody against myelin basic protein (*MBP*; *red*). **a** Coronal sections from the region adjacent to the hematoma, which is to the right but beyond the image (as indicated by “→ H”). The peri-hematoma is visible in the *right*-*most portion* of each image, and the surrounding striatum is visible toward the *left*. Higher-magnification images of the *boxed regions* are shown to the *right*. **b** Sagittal sections. All images were taken in the peri-hematoma, the region immediately adjacent to the hematoma, which is below each portion shown (as indicated by “↓H”). *Scale bars* = 50 μm and apply to all panels

To examine the progression of damage to axons and myelin in a qualitative manner, we used coronal (Fig. 2a) and sagittal (Fig. 2b) sections. Myelinated axon tracts in the striatum arise from excitatory cortical neurons [26]. They were visualized using an antibody against MBP, and axons were labeled with a pan-NF antibody that recognizes the three major neurofilament subunits: light, medium, and heavy chain. The coronal sections show approximately circular myelinated axon tracts extending in the rostral-caudal axis (Fig. 2a). In the saline-injected (control) striatum, bundles are filled with densely packed myelinated axons, as indicated by the abundant overlap of the MBP and pan-NF stains (Fig. 2a). At high magnification, the two stains do not precisely co-localize (also see [27]) because myelin wraps around each neurofilament-containing axon. In sagittal sections from saline-injected animals, there were dense longitudinal tracts of myelinated axons throughout the striatum (Fig. 2b). The sagittal orientation was especially useful for assessing damage to white-matter bundles close to the hematoma.

We previously showed a progressive, dramatic decrease in staining for normal MBP and a concomitant increase in damaged MBP in the peri-hematoma region from 1 to 7 days after ICH [16–18]. In coronal (Fig. 2a) and sagittal sections (Fig. 2b), despite the normal variation in diameter of striatal white-matter tracts, a similar increase in myelin damage is clearly evident over the first week. There was progressive bundle swelling and fragmentation. Interestingly, at 7 days, myelin and axon staining were not well organized into dense bundles, and the high-magnification coronal image shows diffuse neurofilament staining inside fragmented bundles. Beyond the first week, the bundles of myelinated axons appeared denser, less swollen, and less fragmented (i.e., day 14 was similar to day 3). The high-magnification coronal image at 28 days clearly shows that the axons were more aligned into dense myelinated tracts. From the sagittal images, it appears that some bundles were thinner at 14 and 28 days than in the saline control. The improved appearance of the myelinated axon tracts after the first week, and presence of both axons and myelin in the peri-hematoma region throughout the first month after ICH, raises the prospect for re-myelination. Thus, we next determined if oligodendrocyte-lineage cells survived and differentiated into mature cells that are capable of myelin production.

### The Density of Oligodendrocyte-Lineage Cells Increases as a Result of Proliferation in the Peri-hematoma Region

**Fig. 3:**
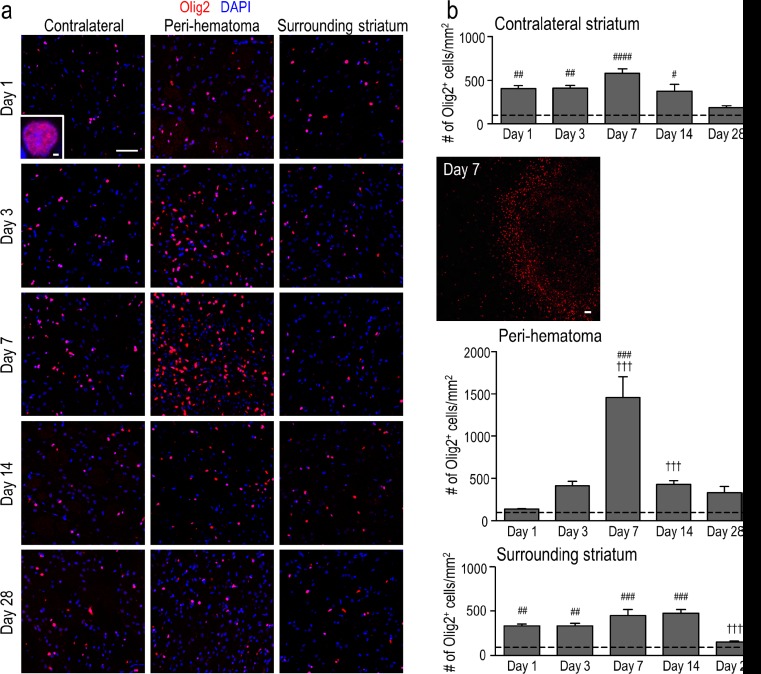
Numbers of oligodendrocyte-lineage cells increase after ICH, especially in the peri-hematoma. **a** Oligodendrocyte-lineage cells of any maturation state were identified using an antibody against the nuclear antigen, Olig2 (*red*). Cell nuclei were stained with DAPI (*blue*), and the *enlarged inset* shows an example of staining co-localization. Representative images from 1 to 28 days after ICH show the contralateral (undamaged) striatum, the peri-hematoma region, and the striatum surrounding the peri-hematoma. *Scale bar* = 50 μm (*main panels*), 10 μm (*inset*). *Inset to right*: Low-magnification image of oligodendrocyte-lineage cells (Olig2; *red*) surrounding the hematoma at 7 days. *Scale bar* = 50 μm. **b** Time-dependent changes in the density of oligodendrocyte-lineage cells were determined by counting double-labeled Olig2^+^DAPI^+^ nuclei. Values represent mean ± SEM from three rats at each time point, and the *dashed line* represents the mean for 1-day saline controls (*n* = 3). Statistical comparisons were based on a one-way ANOVA followed by Tukey’s post-hoc test. Comparisons show differences: † from the preceding time point, # between ICH and 1 day saline controls; one symbol *p* < 0.05, two symbols *p* < 0.01, three symbols *p* < 0.001, four symbols *p* < 0.0001. Additional significant differences that are not illustrated with symbols are as follows: (i) in the peri-hematoma, 7 vs 1 and 28 days; (ii) in the surrounding striatum, 28 vs 1, 3, and 7 days; and (iii) in the contralateral striatum, 28 vs 1, 3, and 7 days

We quantified temporal changes in the density of oligodendrocyte-lineage cells in the peri-hematoma region, surrounding striatum, and undamaged contralateral striatum from 1 to 28 days. All oligodendrocyte-lineage cells, regardless of maturation state, express the transcription factor, Olig2 [28], and, as expected, we found that Olig2 co-localized with the nuclear marker, DAPI (Fig. 3a). It was previously shown that saline injection does not cause damage [15, 25, 29, 30]. As expected, saline-injected controls showed the same density of oligodendrocyte-lineage cells at 1, 3, and 7 days; hence, ICH samples were compared with the 1-day saline control. After ICH, the density of Olig2^+^ cells increased from 1 to 7 days in the peri-hematoma region, at which time they were ∼11-fold higher than controls (Fig. 3b) and formed a dense band around the hematoma (Fig. 3a; inset to right). Their numbers were markedly decreased at 14 and 28 days. In the surrounding striatum further from the hematoma, and in the contralateral striatum, the density of Olig2^+^ cells was elevated at 1, 3, 7, and 14 days and then decreased to the control level at 28 days.

**Fig. 4:**
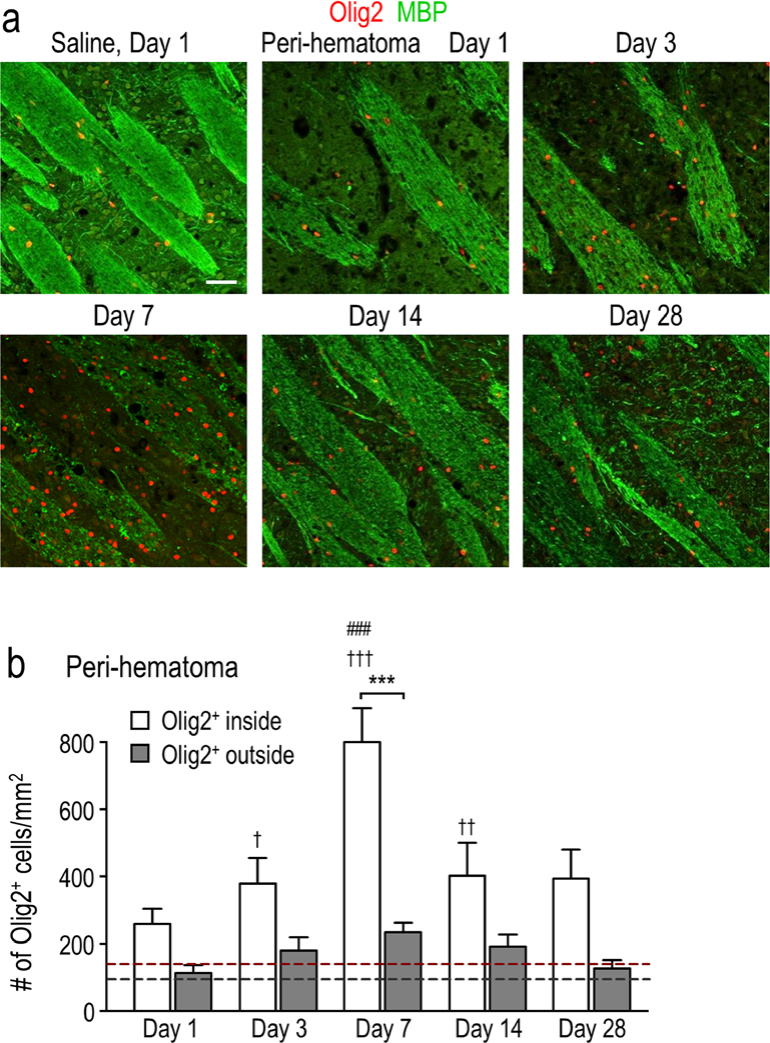
Oligodendrocyte-lineage cells preferentially increase inside white-matter bundles. **a** Representative images from sagittal sections taken from the peri-hematoma region, with myelin bundles labeled with MBP (*green*) and oligodendrocyte-lineage cells of any maturation state labeled with Olig2 (*red*). *Scale bar* = 50 μm and applies to all panels. **b** Density of Olig2^+^ cells inside or outside white-matter bundles in the peri-hematoma region. Values are mean ± SEM from four rats at each time point after ICH. The *dashed lines* indicate mean values for 1-day saline controls (*n* = 3) for oligodendrocyte-lineage cells inside (*red*) and outside bundles (*black*). Statistical comparisons were based on a two-way ANOVA followed by Tukey’s post-hoc test. Comparisons show differences: † from the preceding time point, # from the saline control, * between those inside and outside the bundles; one symbol *p* < 0.05, two symbols *p* < 0.01, three symbols *p* < 0.001

We examined sagittal sections from the peri-hematoma region to ask whether the oligodendrocyte-lineage cells increased inside myelinated white-matter tracts, and in the neuron-rich neuropil, which contains many cell bodies of medium spiny neurons that are damaged after ICH [16–18, 31]. The bundles of myelinated axons were swollen and MBP was degraded at 7 days, as previously shown. As shown in Fig. 2, many myelinated bundles had re-appeared by 14 and 28 days (Fig. 4a). Although Olig2^+^ cells were present both inside and outside the bundles, they preferentially accumulated inside the white-matter tracts (Fig. 4b). Again, their density peaked at 7 days and then decreased. Their density also apparently increased outside the bundles (∼2-fold higher at 7 days) but this did not reach statistical significance.

**Fig. 5:**
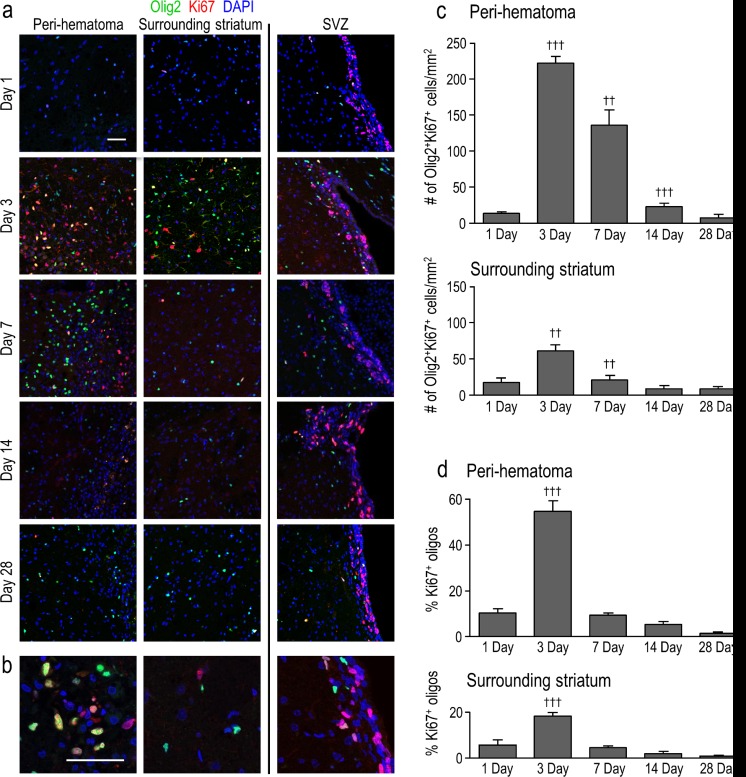
Oligodendrocyte-lineage cells proliferate in the damaged striatum, particularly in the peri-hematoma region. **a** Representative images show oligodendrocyte-lineage cells labeled for Olig2 (*green*), proliferating cells stained with an antibody against Ki67 (*red*), and nuclei labeled with DAPI (*blue*). Images were taken from the ipsilateral side in the peri-hematoma region (*left panels*), surrounding striatum (*middle*), and SVZ (*right*). *Scale bar* = 50 μm and applies to all panels. **b** Higher-magnification images of typical single-, double-, and triple-stained nuclei in each region at 3 days after ICH. **c** Density of proliferating oligodendrocyte-lineage cells (i.e., cells with Olig2^+^Ki67^+^ nuclei) in the peri-hematoma and surrounding striatum from 1 to 28 days after ICH. **d** Percentage of oligodendrocyte-lineage cells that were proliferating. Values are mean ± SEM from three rats at each time. Statistical comparisons were based on a one-way ANOVA followed by Tukey’s post-hoc test and show differences: † from the preceding time point; one symbol *p* < 0.05, two symbols *p* < 0.01, three symbols *p* < 0.001. Additional significant differences in the density of proliferating oligodendrocyte-lineage cells that are not indicated with symbols were as follows: (i) in the peri-hematoma, 1 vs 7 days, 3 vs 14 days, and 28 vs 3 and 7 days; and (ii) in the surrounding striatum, 3 vs 14 and 28 days. For the percentage of oligodendrocyte-lineage cells that were Ki67^+^, all time points were significantly different from 3 days in both the peri-hematoma and surrounding striatum

In principle, the increased density of Olig2^+^ cells observed in the peri-hematoma region could be due to proliferation, migration, or both. To address whether these cells were proliferating within the damaged striatum, we labeled Ki67, a nuclear protein present in all active phases of the cell cycle but not in the G0 phase [32]. Because Olig2 and DAPI co-localize in the nucleus (Fig. 3), we could readily identify proliferating oligodendrocyte-lineage cells as those double-labeled for Olig2 and Ki67. In general, many proliferating cells (Ki67^+^) were present in the peri-hematoma region at 3 and 7 days (Fig. 5a). The higher-magnification images (Fig. 5b) show examples of proliferating (Ki67^+^) Olig2^+^ cells, some oligodendrocyte-lineage cells not in active cell-cycle phases (Ki67^−^Olig2^+^), and some proliferating cells that were not of oligodendrocyte lineage (Ki67^+^Olig2^−^). Proliferating Olig2^+^ cells were then quantified in two ways: as the density of cells double-labeled for Ki67 and as the percentage of Olig2^+^ cells that had re-entered the cell cycle. In the peri-hematoma region, their density increased ∼16-fold between 1 and 3 days and then progressively declined by 14 days to the level seen at 1 day (Fig. 5c). Of note, increased proliferation of Olig2^+^ cells preceded their maximal increase in density at 7 days (Fig. 3). At 3 days, 55 ± 5 % of the Olig2^+^ cells expressed Ki67, while only 9 ± 1 % did so at 7 days (Fig. 5d). In the surrounding striatum, their proliferation showed a similar time course but much lower density, and the proportion of proliferating Olig2^+^ cells was only 18 ± 2 % at 3 days. No proliferating Olig2^+^ cells were detected in the striatum of saline-injected control rats or the contralateral striatum of ICH animals (not shown). Next, we addressed whether proliferating Olig2^+^ cells in the peri-hematoma were likely to have simply migrated from the SVZ, a site of oligodendrogenesis throughout life [33]. While proliferating cells were present in the SVZ at each time point, almost none of them were Olig2^+^ (Fig. 5a, b). Of those few that were double-labeled, their numbers did not change between 1 and 28 days (not shown); thus, proliferation of Olig2^+^ cells at the damage site is apparently important for their increased density.

### OPCs and Mature Oligodendrocytes Increase Transiently in the Peri-hematoma, Preferentially Inside White-Matter Tracts

**Fig. 6:**
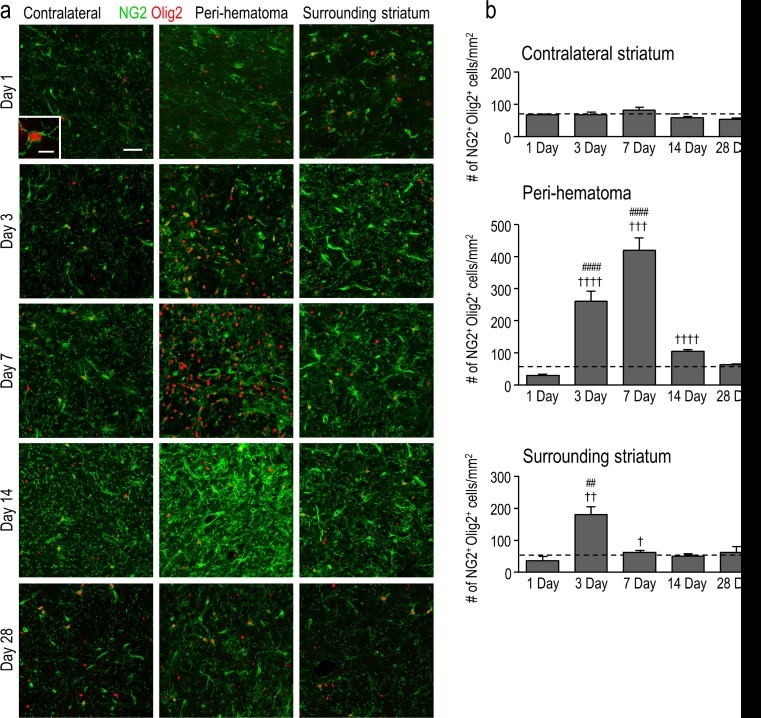

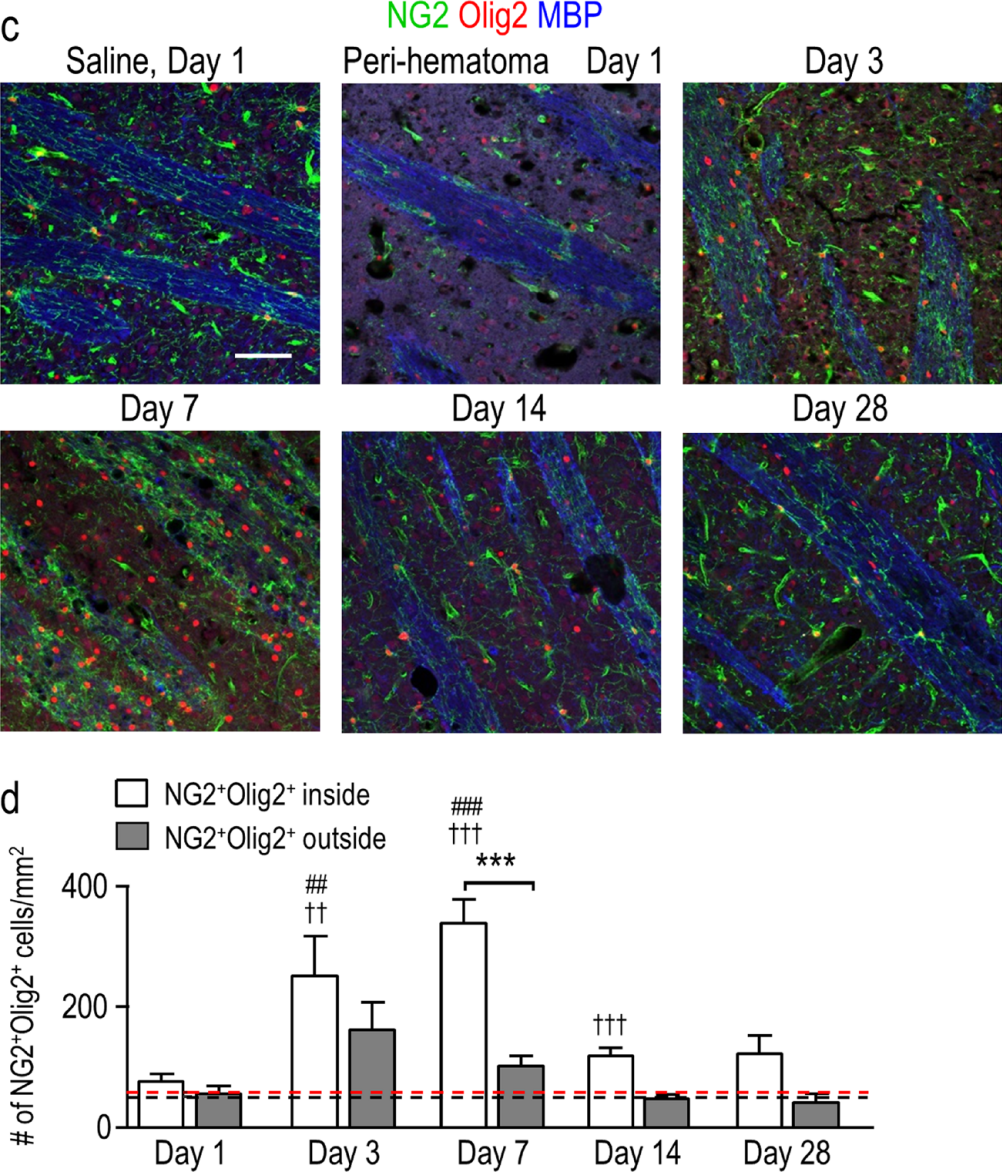
Oligodendrocyte precursor cells (OPCs) increase preferentially in the peri-hematoma and inside white-matter bundles. **a** OPCs were identified as cells with Olig2^+^ nuclei (*red*) that were double-labeled with an antibody against NG2 chondroitin sulfate proteoglycan (*green*). Representative images were taken from the contralateral striatum, and the peri-hematoma region and the surrounding striatum on the ipsilateral side. The higher-magnification *inset* shows a typical OPC, with an Olig2^+^ nucleus surrounded by NG2. *Scale bar* = 50 μm (*main panels*), 10 μm (*inset*). **b** Time-dependent changes in density of OPCs (Olig2^+^NG2^+^ cells). Values represent mean ± SEM from three rats at each time point, and the *dashed line* represents the mean for 1-day saline controls (*n* = 3). Statistical comparisons were based on a one-way ANOVA followed by Tukey’s post-hoc test. Comparisons show differences: † from the preceding time point, # from the 1-day saline control; one symbol *p* < 0.05, two symbols *p* < 0.01, three symbols *p* < 0.001, four symbols *p* < 0.0001. Additional significant differences that are not shown with symbols are as follows: (i) in the peri-hematoma, 7 vs 1 and 28 days, 3 vs 14 and 28 days; and (ii) in the surrounding striatum, 3 vs 14 and 28 days. **c** Representative images from sagittal sections taken from the peri-hematoma region were triple-stained for Olig2 (*red*), NG2 (*green*), and myelin basic protein (MBP; *blue*). *Scale bar* = 50 μm and applies to all panels. **d** Density of OPCs (Olig2^+^NG2^+^ cells) inside or outside white-matter bundles in the peri-hematoma region. Values are mean ± SEM from four rats at each time point after ICH. The *dashed lines* indicate mean levels for 1-day saline controls (*n* = 3) for OPCs inside (*red*) and outside the bundles (*black*). Statistical comparisons were based on a two-way ANOVA, followed by Tukey’s post-hoc test. Comparisons show differences: † from the preceding time point, # from the saline control, * between those inside and outside the bundles; one symbol *p* < 0.05, two symbols *p* < 0.01, three symbols *p* < 0.001

As shown above, the density of oligodendrocyte-lineage cells increased dramatically and peaked at 7 days, at which time many were inside the damaged white-matter bundles (Figs. 3 and 4). However, in order to generate and repair myelin, they must undergo maturation [34]; therefore, we next quantified their maturation states. Immature oligodendrocyte precursor cells (OPCs) were identified as cells with Olig2^+^ nuclei that also stained for NG2 chondroitin sulfate proteoglycan [35]. It was necessary to double-label the cells (see high-magnification image in Fig. 6a) because activated microglia and macrophages can also express NG2 [36]. In the undamaged contralateral striatum, there was no change in the density of OPCs (NG2^+^Olig2^+^) compared with saline controls (Fig. 6a, b). In contrast, in the peri-hematoma region, their density increased dramatically at 3 and 7 days (∼8-fold at 7 days) and then decreased, reaching control levels by 28 days. Sagittal sections showed a similar time-dependent increase in OPCs, preferentially inside the white-matter bundles (Fig. 6c, d). In the surrounding striatum, there was a much smaller increase, with statistical significance only at 3 days (Fig. 6b).

**Fig. 7:**
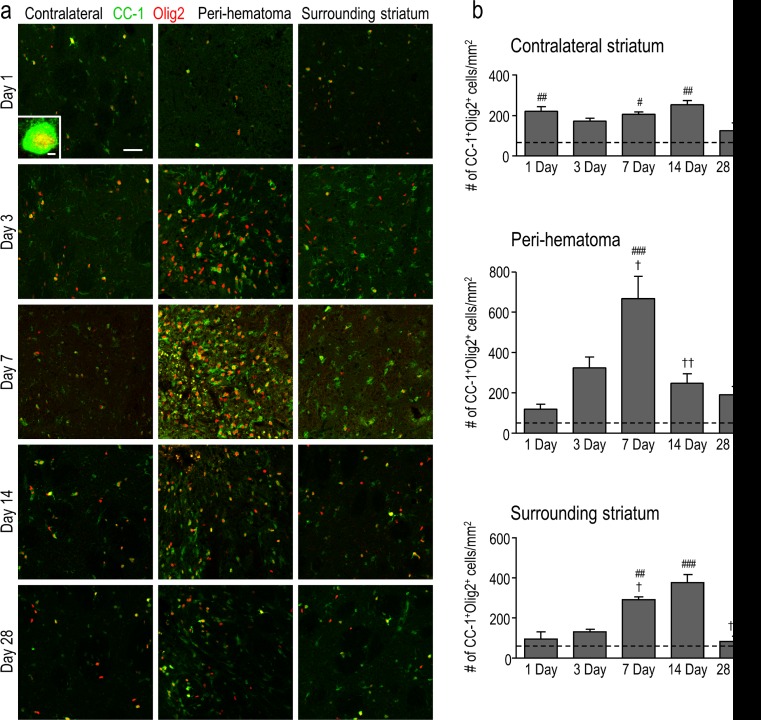

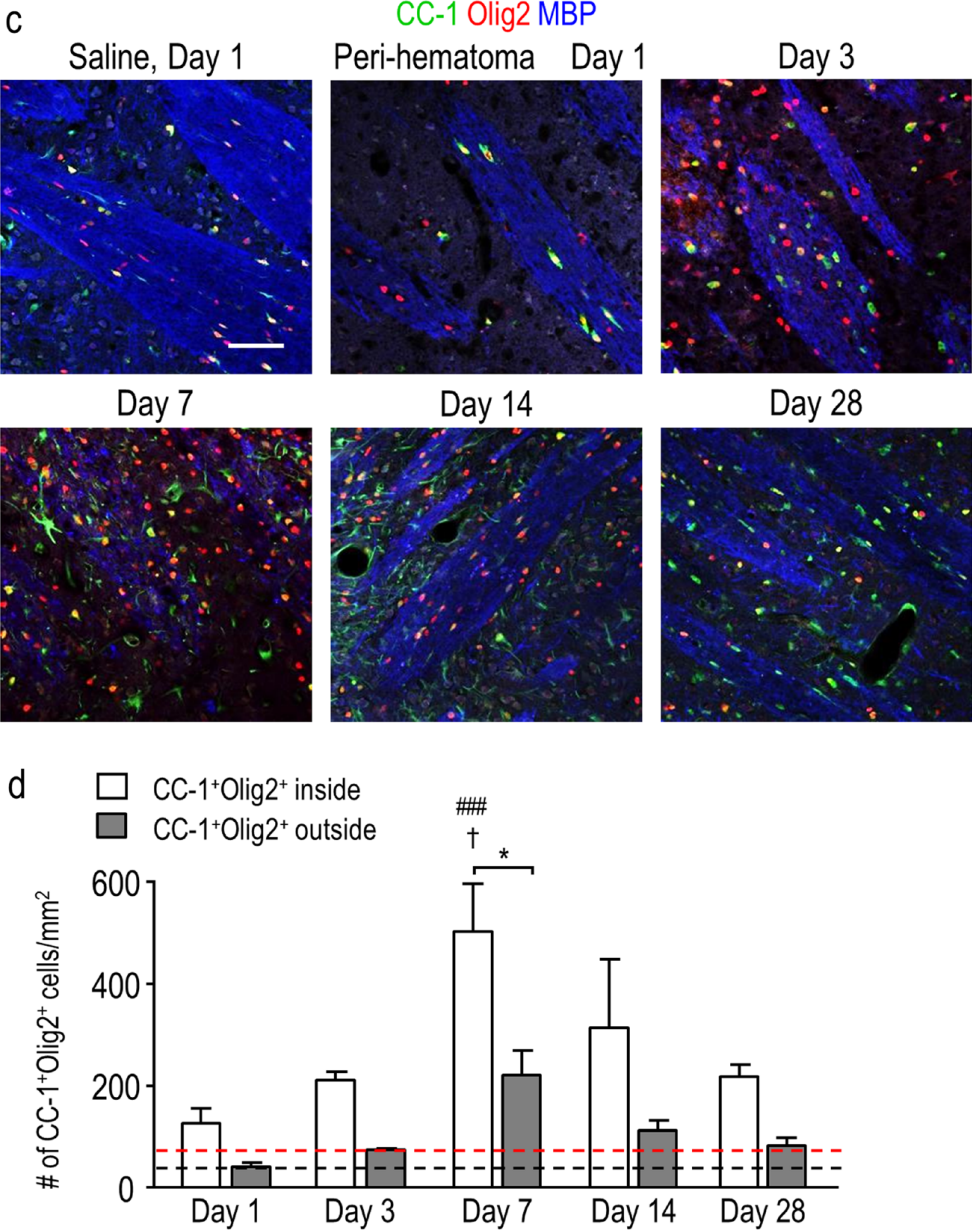
Mature oligodendrocytes increase after ICH, especially inside white-matter bundles in the peri-hematoma. **a** Mature oligodendrocytes were identified as cells with Olig2^+^ nuclei (*red*) that were double-labeled with an antibody against *adenomatous polyposis coli* (APC, also called CC-1; *green*). Representative images were taken from the contralateral striatum, and the peri-hematoma and surrounding striatum on the ipsilateral side. The higher-magnification *inset* shows a typical mature oligodendrocyte with an Olig2^+^ nucleus surrounded by CC-1. *Scale bar* = 50 μm (*main panels*), 10 μm (*inset*). **b** Time-dependent changes by density of mature oligodendrocytes (Olig2^+^CC-1^+^ cells). Values represent mean ± SEM from three rats at each time point, and the *dashed line* represents the mean for 1-day saline controls (*n* = 3). Statistical comparisons were based on a one-way ANOVA followed by Tukey’s post-hoc test. Comparisons show differences: † from the preceding time point, # from the 1-day saline control; one symbol *p* < 0.05, two symbols *p* < 0.01, three symbols *p* < 0.001. Additional significant differences that are not shown with symbols are as follows: (i) in the peri-hematoma, 7 vs 1 and 28 days; and (ii) in the surrounding striatum, 7 vs 1 and 28 days and 14 vs 1 and 3 days. **c** Representative images from sagittal sections from the peri-hematoma region that were triple-stained for Olig2 (*red*), CC-1 (*green*), and myelin basic protein (MBP; *blue*). *Scale bar* = 50 μm and applies to all panels. **d** Density of mature oligodendrocytes (Olig2^+^/CC-1^+^) inside or outside white-matter bundles in the peri-hematoma region. Values are mean ± SEM from four rats at each time point. The *dashed lines* indicate mean values for 1-day saline controls (*n* = 3) for oligodendrocytes inside (*red*) and outside the bundles (*black*). Statistical comparisons were based on a two-way ANOVA, followed by Tukey’s post-hoc test. Comparisons show differences: † from the preceding time point, # from the saline control, * between those inside and outside the bundles; one symbol *p* < 0.05, two symbols *p* < 0.01, three symbols *p* < 0.001

To quantify the density of mature oligodendrocytes, cells were double-labeled for Olig2 and CC-1. CC-1 labels *adenomatous polyposis coli* (APC), a tumor suppressor protein that is present in the cell body of mature oligodendrocytes and thought to regulate their adhesive properties [37]. Double-labeling for CC-1 and Olig2 is illustrated in the high-magnification inset in Fig. 7a. In the peri-hematoma region, the density of mature oligodendrocytes increased with time, reached a peak at 7 days that was ∼7-fold higher than that of saline controls (Fig. 7a, b), and then declined. This time-dependent profile was similar to that of total oligodendrocyte-lineage cells (Fig. 3b) and OPCs (Fig. 6b), but the density of mature cells was higher than that of OPCs. In the surrounding striatum, mature oligodendrocytes increased at 7 and 14 days and then decreased to control levels at 28 days. In the undamaged contralateral striatum, the density of mature oligodendrocytes was increased by 2–3-fold as early as 1 day and remained elevated for at least 14 days (Fig. 7a, b). Because there was no increase in immature OPCs (Fig. 6b) or proliferating Olig2^+^ cells, this suggests that the cells migrated into the striatum from other regions soon after ICH. Finally, mature oligodendrocytes increased preferentially inside the white-matter bundles (Fig. 7c, d), as was shown for total oligodendrocyte-lineage cells (Fig. 4) and OPCs (Fig. 6). However, the greatest increases in mature cells were at 7 and 14 days (Fig. 7) compared with 3 and 7 days for OPCs (Fig. 6). Together with the proliferation data (Fig. 5), these observations provide evidence for local replenishment of both immature OPCs and mature oligodendrocytes in the peri-hematoma region, and preferentially inside white-matter tracts.

## Discussion

White-matter damage often occurs after acute CNS injury [38] and is considered an important predictor of the outcome, including after stroke [39, 40]. After intracerebral hemorrhage (ICH), significant white-matter damage was seen in CT scans of 77 % of patients [9] and large white-matter lesions were associated with severe Glasgow Coma Scale scores [40]. MRI showed that white-matter hyper-intensity volumes were higher after ICH than after ischemic strokes [41]. In most experimental stroke studies that consider white matter, the focus has been on ischemia and on treatment targets ranging from reducing immediate damage to promoting endogenous repair/regeneration to replacing cells using exogenous sources. In evaluating approaches for repairing white matter, it is important to know the fate of axons and oligodendrocytes but almost nothing is known about these aspects after ICH. We found only one previous study, which showed that after experimental ICH in the mouse cortex, staining of mature oligodendrocytes with RIP antibody (which recognizes 2′,3′-cyclic nucleotide 3′-phosphodiesterase; CNPase) decreased at 7 days [42]. The location with respect to the hematoma was not stated, and temporal changes, proliferation, and differentiation of oligodendrocyte-lineage cells were not addressed. The present study appears to be the first to address the fate of oligodendrocyte-lineage cells after ICH in the rat. We focused on the peri-hematoma region and surrounding striatum because tissue is immediately damaged inside the hematoma and is unlikely to be salvageable [43]. We will discuss each aspect in light of the limited literature.

We used coronal and sagittal sections to assess white-matter tracts that are comprised of myelinated corticostriatal axons. In this model, we previously quantified a progressive loss of normal myelin and accumulation of damaged myelin in the first week [16, 17, 27]. Here, similar progressive myelin damage was seen but a new finding was that both axons and oligodendrocytes survived in the peri-hematoma region and surrounding striatum for at least 28 days, the longest time tested. Initially, as white-matter damage progressed during the first week, the myelinated tracts in the peri-hematoma region became swollen, which is consistent with previously identified white-matter edema [44]. The bundles became increasingly fragmented during the first week but still recognizable, and extensive, diffuse neurofilament staining remained at 7 days. In earlier ICH studies, we observed transient accumulation of amyloid precursor protein (APP) and SC1/hevin within axons in the peri-hematoma at 1 and 3 days [16, 17, 27]. At the time, we noted that accumulation of these molecules indicates impaired axoplasmic transport but does not mean the axons are irreversibly damaged. We had not previously examined times beyond 7 days and were surprised to find that at 14 and 28 days, many discrete myelinated bundles of axons were again present and appeared normal. While it is possible that severely fragmented tracts had been removed, there was no apparent decrease in the number of bundles compared with the undamaged striatum. Instead, in sagittal sections, some bundles appeared thinner (a characteristic of regenerated myelin [45]), and this is consistent with an observation at 100 days after autologous blood injection into the rat striatum [46]. An earlier ICH study in mice reported fewer corticostriatal axons at 7 days, but an increase at 9 weeks [47].

The present finding that axon bundles were poorly organized at 7 days but well organized at 14 and 28 days suggests that some recovery had occurred. While a large battery of tests is needed to assess neurological outcomes after damage to the striatum [48], several studies of collagenase-induced ICH in the rat show varying degrees of spontaneous recovery. Over several weeks, ICH animals exhibited recovery in several motor and cognitive tasks [48–51] but still had some sensorimotor deficits up to 8 weeks [50, 51]. Not surprisingly, greater recovery is generally seen for small- to medium-sized lesions [49]: the size range in the present study. In the most similar study, motor deficits had resolved at 30 days, as judged by the composite neurological deficit scale, which assesses spontaneous ipsilateral circling, hind limb retraction, bilateral forepaw grasp, beam walking ability, and forelimb flexion [48].

The main emphasis of this study was on the fate of oligodendrocyte-lineage cells. In the peri-hematoma region, both immature OPCs and mature oligodendrocytes were increased substantially from 1 to 7 days and preferentially enriched inside white-matter bundles. They were also increased throughout the surrounding striatum. In both locations, their density then declined from 14 to 28 days. By 7 days, both immature OPCs and mature oligodendrocytes formed a ring around the hematoma, with a similar distribution to the peri-hematoma ring of activated microglia/macrophages [17]. Moreover, the immune cells were preferentially inside white-matter bundles that labeled for damaged myelin basic protein. This raises important questions for future study. Are activated microglia/macrophages phagocytosing myelin debris to promote re-myelination? Do they initially support survival and proliferation of oligodendrocyte-lineage cells and then, at later times, kill and remove excess cells? Another possibility is that the decline in oligodendrocyte-lineage cells at later times results from toxic effects of blood components. In vitro, blood proteins can suppress migration of OPCs and thrombin can suppress OPC differentiation [52]. Iron liberated from ruptured erythrocytes causes oxidative stress [24, 53], and iron remains elevated for at least 1 month in the collagenase ICH model [24, 54].

In principle, the increase in the density of oligodendrocyte-lineage cells in the peri-hematoma and surrounding striatum after ICH could be due to proliferation, migration from other areas, or both. In the present study, the striatum contained many proliferating oligodendrocyte-lineage cells but very few were seen in the subventricular zone (SVZ). Similarly, in rodent models of ischemic stroke, proliferating NG2^+^ OPCs were also prevalent in the peri-infarct region, maximal at 7 days [55, 56], and then decreased at 28 days [56]. The SVZ is considered a source of neural progenitor cells that migrate out and can differentiate into oligodendrocytes, but previous ICH studies have investigated the SVZ only from a neurogenesis perspective [57–62]. The site of oligodendrogenesis might also depend on the disease model and where the damage is located. For instance, in Huntington’s disease, oligodendrocyte-lineage cells proliferated within the striatum [63] but not in the SVZ [63–66]. After ischemic stroke (murine middle cerebral artery occlusion model), fate-mapping showed some SVZ-derived oligodendrocytes in the striatum [67, 68]. After de-myelination of the mouse corpus callosum, OPC proliferation was seen within the SVZ [33, 69].

The oligodendrocyte density also increased in the contralateral striatum from 1 to 14 days, but we did not detect proliferating OPCs at any time. In the future, with optical tagging or genetic markers, it might be possible to determine whether their source is within the striatum or from the corpus callosum, SVZ, or another location. We could not find previous publications addressing oligodendrocyte responses in the contralateral side after ICH. However, there are numerous indications that the contralateral hemisphere does respond after ICH, i.e., changes in gene expression [18, 70], enhanced dendritic arborization [23], loss of corticostriatal axons [47], and increased axial diffusivity [71].

The substantial increase in OPC proliferation suggests that ICH upregulates mitogens in the peri-hematoma and surrounding striatum. There are several good candidates. Insulin-like growth factor-1 (IGF-1) stimulates OPC proliferation in vitro [72]. Platelet-derived growth factor (PDGF) activates the α receptor (PDGFRα) on OPCs and increases their proliferation and survival in vitro [73–75] and in organotypic slices [76]. Fibroblast growth factor-2 (FGF-2) stimulates OPC proliferation [77] and upregulates their expression of PDGFRα, which prevents them from maturing and, thus, prolongs their responses to PDGF [78]. In the future, it would be interesting to examine the spatial and temporal expression of these potential mitogens after ICH. There appears to be very limited information about their expression after ICH, with data from two microarray studies. Within 24 h after ICH, FGF-2 was ∼1.5-fold lower in the peri-hematoma region of four ICH patients [79], and the FGF-2 receptor, FGF2rb, was ∼2-fold lower in the striatum and cortex in the rat autologous blood-injection model [80]. Interestingly, FGF-2 supplementation reduced edema and improved some motor functions in the collagenase- and autologous-blood injection ICH models in mice [81].

Mature oligodendrocytes are required for re-myelination [82]; thus, an important finding was that more than half the oligodendrocyte-lineage cells in the ipsilateral striatum were mature (CC-1^+^) by 7 days. This time course is consistent with a 3- to 4-day period for NG2^+^ OPCs to differentiate into mature CC-1^+^ oligodendrocytes after acute demyelination in forebrain slice cultures [83]. CC-1 does not distinguish if these cells are actively myelinating. Future studies will need to examine myelination markers and re-myelination itself. Our results suggest that differentiation factors are present early after ICH, and there are several good candidates. Brain-derived neurotrophic factor (BDNF) can stimulate OPC differentiation in vitro, during development and after white-matter ischemia in mice [84–87], and after subcortical ischemia in rats [88]. Other potential OPC differentiation factors are transforming growth factor beta (TGF-β) [89], IGF-1 [72, 90], thyroid hormone [82], and epidermal growth factor (EGF) [91, 92]. Microglia can produce these and other mediators that promote oligodendrocyte proliferation and differentiation in vitro and in other demyelination models in vivo [93–96]. Again, there is limited data on expression of these molecules after ICH. Most of the published data concern TGF-β and BDNF. In the first week after collagenase-induced ICH in the rat, the damaged striatum showed increases in transcript expression for TGF-β [18, 70] and BDNF (S Lively and LC Schlichter, unpublished results). Microarray data from peri-hematomal tissue of four ICH patients showed a nearly 3-fold increase in TGF-β transcripts in the first day [97]. In the rat autologous blood-injection model, BDNF protein did not change in the peri-hematoma at 7 days [98]. Clearly, future studies will be needed to determine the spatiotemporal expression, cellular sources, and contributions of potential oligodendrocyte differentiation factors after ICH.

### Summary and Conclusions

Strategies to treat white-matter injury focus on reducing oligodendrocyte loss, adding growth factors to promote their proliferation, maturation, and re-myelination, or supplementing with exogenous stem cells [99, 100]. Although stem cell replacement is used in experimental studies of ischemic stroke, and injected mesenchymal stem cells can differentiate into oligodendrocytes (as well as neurons and astrocytes [101]), there is surprisingly little evidence supporting any of these approaches after ICH. This is apparently the first study to examine and quantify the spatial and temporal distribution of oligodendrocyte-lineage cells, and their proliferation and maturation after experimental ICH. There are several salient findings. (i) Axons and oligodendrocytes survived for at least a month in the peri-hematoma region and surrounding striatum, and by 2 weeks, there were signs that the myelinated corticostriatal axon tracts were recovering. (ii) The density of both immature OPCs and mature oligodendrocytes increased dramatically in the peri-hematoma region for the first week and then declined to about control levels. Their source was likely local because considerable proliferation of oligodendrocyte-lineage cells was seen in the peri-hematoma and surrounding striatum (peak at 3 days) but not in the subventricular zone during the first month. (iii) Both immature OPCs and mature oligodendrocytes were present throughout the first month, and when their density peaked at about 1 week, they were preferentially located inside white-matter bundles. Thus, endogenous cells that are required for re-myelination are present in the right place for the first month after ICH. These results provide useful benchmarks for assessing potential therapies to rescue the tissue surrounding the hemorrhage, for further investigating the cellular basis of damage after ICH, and for improving white-matter recovery and function. Future studies will need to address whether the new oligodendrocytes are essential for re-myelinating axons and whether these axons are functionally integrated into the proper circuitry.

## Abbreviations

ICH: Intracerebral hemorrhage
OPC: Oligodendrocyte precursor cell
PBS: Phosphate-buffered saline
MBP: Myelin basic protein
pan-NF: Pan-axonal neurofilament
Olig2: Oligodendrocyte lineage transcription factor 2
NG2: Neural/glial antigen 2
APC: Adenomatous polyposis coli
DAPI: 4′-6-Diamidino-2-phenylindole
SVZ: Subventricular zone
ANOVA: Analysis of variance
CSF: Cerebrospinal fluid
CNPase: 2′,3′-Cyclic nucleotide 3′-phosphodiesterase
APP: Amyloid precursor protein
IGF-1: Insulin-like growth factor-1
PDGF: Platelet-derived growth factor
PDGFRα: Platelet-derived growth factor receptor alpha
FGF-2: Fibroblast growth factor-2
BDNF: Brain-derived neurotrophic factor
TGF-β: Transforming growth factor beta
EGF: Epidermal growth factor

## References

[1] Feigin VL, Lawes CM, Bennett DA, Barker-Collo SL, Parag V (2009). Worldwide stroke incidence and early case fatality reported in 56 population-based studies: a systematic review. Lancet Neurol.

[2] van Asch CJ, Luitse MJ, Rinkel GJ, van der Tweel I, Algra A, Klijn CJ (2010). Incidence, case fatality, and functional outcome of intracerebral haemorrhage over time, according to age, sex, and ethnic origin: a systematic review and meta-analysis. Lancet Neurol.

[3] Poon MT, Fonville AF, Al-Shahi SR (2014). Long-term prognosis after intracerebral haemorrhage: systematic review and meta-analysis. J Neurol Neurosurg Psychiatry.

[4] Hemphill JC, Greenberg SM, Anderson CS, Becker K, Bendok BR, Cushman M (2015). Guidelines for the management of spontaneous intracerebral hemorrhage: a guideline for healthcare professionals from the American Heart Association/American Stroke Association. Stroke; J Cereb Circ.

[5] Anderson CS (2009). Medical management of acute intracerebral hemorrhage. Curr Opin Crit Care.

[6] Sangha N, Gonzales NR (2011). Treatment targets in intracerebral hemorrhage. Neurotherapeutics.

[7] Qureshi AI, Tuhrim S, Broderick JP, Batjer HH, Hondo H, Hanley DF (2001). Spontaneous intracerebral hemorrhage. N Engl J Med.

[8] Keep RF, Hua Y, Xi G (2012). Intracerebral haemorrhage: mechanisms of injury and therapeutic targets. Lancet Neurol.

[9] Smith EE, Gurol ME, Eng JA, Engel CR, Nguyen TN, Rosand J (2004). White matter lesions, cognition, and recurrent hemorrhage in lobar intracerebral hemorrhage. Neurology.

[10] Kellner CP, Connolly ES (2010). Neuroprotective strategies for intracerebral hemorrhage: trials and translation. Stroke; Jo Cereb Circ.

[11] MacLellan CL, Paquette R, Colbourne F (2012). A critical appraisal of experimental intracerebral hemorrhage research. J Cereb Blood Flow Metab.

[12] Petty MA, Wettstein JG (1999). White matter ischaemia. Brain Res Brain Res Rev.

[13] Stys PK, Lipton SA (2007). White matter NMDA receptors: an unexpected new therapeutic target?. Trends Pharmacol Sci.

[14] Xi G, Keep RF, Hoff JT (2006). Mechanisms of brain injury after intracerebral haemorrhage. Lancet Neurol.

[15] Wasserman JK, Yang H, Schlichter LC (2008). Glial responses, neuron death and lesion resolution after intracerebral hemorrhage in young vs. aged rats. Eur J Neurosc.

[16] Wasserman JK, Schlichter LC (2008). White matter injury in young and aged rats after intracerebral hemorrhage. Exp Neurol.

[17] Moxon-Emre I, Schlichter LC (2011). Neutrophil depletion reduces blood-brain barrier breakdown, axon injury, and inflammation after intracerebral hemorrhage. J Neuropathol Exp Neurol.

[18] Lively S, Schlichter LC (2012). Age-related comparisons of evolution of the inflammatory response after intracerebral hemorrhage in rats. Transl Stroke Res.

[19] Crapo PM, Gilbert TW, Badylak SF (2011). An overview of tissue and whole organ decellularization processes. Biomaterials.

[20] Sirkin DW (1983). Critical defatting of frozen brain sections for optimal differentiation with the cresyl violet stain. Stain Technol.

[21] Del Bigio MR (1993). Neuropathological changes caused by hydrocephalus. Acta Neuropathol.

[22] MacLellan CL, Silasi G, Poon CC, Edmundson CL, Buist R, Peeling J (2008). Intracerebral hemorrhage models in rat: comparing collagenase to blood infusion. J Cereb Blood Flow Metab.

[23] Nguyen AP, Huynh HD, Sjovold SB, Colbourne F (2008). Progressive brain damage and alterations in dendritic arborization after collagenase-induced intracerebral hemorrhage in rats. Curr Neurovasc Res.

[24] Auriat AM, Silasi G, Wei Z, Paquette R, Paterson P, Nichol H (2012). Ferric iron chelation lowers brain iron levels after intracerebral hemorrhage in rats but does not improve outcome. Exp Neurol.

[25] Caliaperumal J, Ma Y, Colbourne F (2012). Intra-parenchymal ferrous iron infusion causes neuronal atrophy, cell death and progressive tissue loss: implications for intracerebral hemorrhage. Exp Neurol.

[26] Calabresi P, Pisani A, Mercuri NB, Bernardi G (1996). The corticostriatal projection: from synaptic plasticity to dysfunctions of the basal ganglia. Trends Neurosci.

[27] Lively S, Schlichter LC (2012). SC1/hevin identifies early white matter injury after ischemia and intracerebral hemorrhage in young and aged rats. J Neuropathol Exp Neurol.

[28] Zhou Q, Wang S, Anderson DJ (2000). Identification of a novel family of oligodendrocyte lineage-specific basic helix-loop-helix transcription factors. Neuron.

[29] Wasserman JK, Schlichter LC (2007). Minocycline protects the blood-brain barrier and reduces edema following intracerebral hemorrhage in the rat. Exp Neurol.

[30] DeBow SB, Davies ML, Clarke HL, Colbourne F (2003). Constraint-induced movement therapy and rehabilitation exercises lessen motor deficits and volume of brain injury after striatal hemorrhagic stroke in rats. Stroke; J Cereb Circ.

[31] Wasserman JK, Schlichter LC (2007). Neuron death and inflammation in a rat model of intracerebral hemorrhage: effects of delayed minocycline treatment. Brain Res.

[32] Scholzen T, Gerdes J (2000). The Ki-67 protein: from the known and the unknown. J Cell Physiol.

[33] Menn B, Garcia-Verdugo JM, Yaschine C, Gonzalez-Perez O, Rowitch D, Alvarez-Buylla A (2006). Origin of oligodendrocytes in the subventricular zone of the adult brain. J Neurosci.

[34] Itoh K, Maki T, Lok J, Arai K (2015). Mechanisms of cell-cell interaction in oligodendrogenesis and remyelination after stroke. Brain Res.

[35] Kitada M, Rowitch DH (2006). Transcription factor co-expression patterns indicate heterogeneity of oligodendroglial subpopulations in adult spinal cord. Glia.

[36] Zhu L, Xiang P, Guo K, Wang A, Lu J, Tay SS (2012). Microglia/monocytes with NG2 expression have no phagocytic function in the cortex after LPS focal injection into the rat brain. Glia.

[37] Bhat RV, Axt KJ, Fosnaugh JS, Smith KJ, Johnson KA, Hill DE (1996). Expression of the APC tumor suppressor protein in oligodendroglia. Glia.

[38] Matute C, Ransom BR (2012). Roles of white matter in central nervous system pathophysiologies. ASN Neuro.

[39] Medana IM, Esiri MM (2003). Axonal damage: a key predictor of outcome in human CNS diseases. Brain.

[40] Lee SH, Kim BJ, Ryu WS, Kim CK, Kim N, Park BJ (2010). White matter lesions and poor outcome after intracerebral hemorrhage: a nationwide cohort study. Neurology.

[41] Rost NS, Rahman RM, Biffi A, Smith EE, Kanakis A, Fitzpatrick K (2010). White matter hyperintensity volume is increased in small vessel stroke subtypes. Neurology.

[42] Masuda T, Maki M, Hara K, Yasuhara T, Matsukawa N, Yu S (2010). Peri-hemorrhagic degeneration accompanies stereotaxic collagenase-mediated cortical hemorrhage in mouse. Brain Res.

[43] Ziai WC (2013). Hematology and inflammatory signaling of intracerebral hemorrhage. Stroke; J Cereb Circ.

[44] Del Bigio MR, Yan HJ, Buist R, Peeling J (1996). Experimental intracerebral hemorrhage in rats. Magnetic resonance imaging and histopathological correlates. Stroke; J Cereb Circ.

[45] Gledhill RF, McDonald WI (1977). Morphological characteristics of central demyelination and remyelination: a single-fiber study. Ann Neurol.

[46] Felberg RA, Grotta JC, Shirzadi AL, Strong R, Narayana P, Hill-Felberg SJ (2002). Cell death in experimental intracerebral hemorrhage: the "black hole" model of hemorrhagic damage. Ann Neurol.

[47] Barratt HE, Lanman TA, Carmichael ST (2014). Mouse intracerebral hemorrhage models produce different degrees of initial and delayed damage, axonal sprouting, and recovery. J Cereb Blood Flow Metab.

[48] MacLellan CL, Langdon KD, Churchill KP, Granter-Button S, Corbett D (2009). Assessing cognitive function after intracerebral hemorrhage in rats. Behav Brain Res.

[49] MacLellan CL, Auriat AM, McGie SC, Yan RH, Huynh HD, De Butte MF (2006). Gauging recovery after hemorrhagic stroke in rats: implications for cytoprotection studies. J Cereb Blood Flow Metab.

[50] Hartman R, Lekic T, Rojas H, Tang J, Zhang JH (2009). Assessing functional outcomes following intracerebral hemorrhage in rats. Brain Res.

[51] Beray-Berthat V, Delifer C, Besson VC, Girgis H, Coqueran B, Plotkine M (2010). Long-term histological and behavioural characterisation of a collagenase-induced model of intracerebral haemorrhage in rats. J Neurosci Methods.

[52] Juliet PA, Frost EE, Balasubramaniam J, Del Bigio MR (2009). Toxic effect of blood components on perinatal rat subventricular zone cells and oligodendrocyte precursor cell proliferation, differentiation and migration in culture. J Neurochem.

[53] Wu H, Wu T, Xu X, Wang J, Wang J (2011). Iron toxicity in mice with collagenase-induced intracerebral hemorrhage. J Cereb Blood Flow Metab.

[54] Caliaperumal J, Colbourne F (2014). Rehabilitation improves behavioral recovery and lessens cell death without affecting iron, ferritin, transferrin, or inflammation after intracerebral hemorrhage in rats. Neurorehabil Neural Repair.

[55] Ohta K, Iwai M, Sato K, Omori N, Nagano I, Shoji M (2003). Dissociative increase of oligodendrocyte progenitor cells between young and aged rats after transient cerebral ischemia. Neurosci Lett.

[56] Sozmen EG, Kolekar A, Havton LA, Carmichael ST (2009). A white matter stroke model in the mouse: axonal damage, progenitor responses and MRI correlates. J Neurosci Methods.

[57] Masuda T, Isobe Y, Aihara N, Furuyama F, Misumi S, Kim TS (2007). Increase in neurogenesis and neuroblast migration after a small intracerebral hemorrhage in rats. Neurosci Lett.

[58] Yang S, Song S, Hua Y, Nakamura T, Keep RF, Xi G (2008). Effects of thrombin on neurogenesis after intracerebral hemorrhage. Stroke; J Cereb Circ.

[59] Yan YP, Lang BT, Vemuganti R, Dempsey RJ (2009). Persistent migration of neuroblasts from the subventricular zone to the injured striatum mediated by osteopontin following intracerebral hemorrhage. J Neurochem.

[60] Xu X, Zhang J, Chen X, Liu J, Lu H, Yang P (2012). The increased expression of metabotropic glutamate receptor 5 in subventricular zone neural progenitor cells and enhanced neurogenesis in a rat model of intracerebral hemorrhage. Neuroscience.

[61] Otero L, Zurita M, Bonilla C, Rico MA, Aguayo C, Rodriguez A (2012). Endogenous neurogenesis after intracerebral hemorrhage. Histol Histopathol.

[62] Kang K, Kim YJ, Lee SH, Yoon BW (2014). Lithium fails to enhance neurogenesis in subventricular zone and dentate subgranular zone after intracerebral hemorrhage in rats. Neurol Res.

[63] McCollum MH, Leon RT, Rush DB, Guthrie KM, Wei J (2013). Striatal oligodendrogliogenesis and neuroblast recruitment are increased in the R6/2 mouse model of Huntington's disease. Brain Res.

[64] Lazic SE, Grote HE, Blakemore C, Hannan AJ, van Dellen A, Phillips W (2006). Neurogenesis in the R6/1 transgenic mouse model of Huntington's disease: effects of environmental enrichment. Eur J Neurosci.

[65] Kohl Z, Regensburger M, Aigner R, Kandasamy M, Winner B, Aigner L (2010). Impaired adult olfactory bulb neurogenesis in the R6/2 mouse model of Huntington's disease. BMC Neurosci.

[66] Simpson JM, Gil-Mohapel J, Pouladi MA, Ghilan M, Xie Y, Hayden MR (2011). Altered adult hippocampal neurogenesis in the YAC128 transgenic mouse model of Huntington disease. Neurobiol Dis.

[67] Li L, Harms KM, Ventura PB, Lagace DC, Eisch AJ, Cunningham LA (2010). Focal cerebral ischemia induces a multilineage cytogenic response from adult subventricular zone that is predominantly gliogenic. Glia.

[68] Zhang RL, Chopp M, Roberts C, Jia L, Wei M, Lu M (2011). Ascl1 lineage cells contribute to ischemia-induced neurogenesis and oligodendrogenesis. J Cereb Blood Flow Metab.

[69] Xing YL, Roth PT, Stratton JA, Chuang BH, Danne J, Ellis SL (2014). Adult neural precursor cells from the subventricular zone contribute significantly to oligodendrocyte regeneration and remyelination. J Neurosci.

[70] Wasserman JK, Zhu X, Schlichter LC (2007). Evolution of the inflammatory response in the brain following intracerebral hemorrhage and effects of delayed minocycline treatment. Brain Res.

[71] Fan SJ, Lee FY, Cheung MM, Ding AY, Yang J, Ma SJ (2013). Bilateral substantia nigra and pyramidal tract changes following experimental intracerebral hemorrhage: an MR diffusion tensor imaging study. NMR Biomed.

[72] McMorris FA, Dubois-Dalcq M (1988). Insulin-like growth factor I promotes cell proliferation and oligodendroglial commitment in rat glial progenitor cells developing in vitro. J Neurosci Res.

[73] Noble M, Murray K, Stroobant P, Waterfield MD, Riddle P (1988). Platelet-derived growth factor promotes division and motility and inhibits premature differentiation of the oligodendrocyte/type-2 astrocyte progenitor cell. Nature.

[74] Barres BA, Hart IK, Coles HS, Burne JF, Voyvodic JT, Richardson WD (1992). Cell death and control of cell survival in the oligodendrocyte lineage. Cell.

[75] Grinspan JB, Franceschini B (1995). Platelet-derived growth factor is a survival factor for PSA-NCAM+ oligodendrocyte pre-progenitor cells. J Neurosci Res.

[76] Hill RA, Patel KD, Medved J, Reiss AM, Nishiyama A (2013). NG2 cells in white matter but not gray matter proliferate in response to PDGF. J Neurosci.

[77] Grinspan JB, Stern JL, Franceschini B, Pleasure D (1993). Trophic effects of basic fibroblast growth factor (bFGF) on differentiated oligodendroglia: a mechanism for regeneration of the oligodendroglial lineage. J Neurosci Res.

[78] McKinnon RD, Matsui T, Dubois-Dalcq M, Aaronson SA (1990). FGF modulates the PDGF-driven pathway of oligodendrocyte development. Neuron.

[79] Rosell A, Vilalta A, Garcia-Berrocoso T, Fernandez-Cadenas I, Domingues-Montanari S, Cuadrado E (2011). Brain perihematoma genomic profile following spontaneous human intracerebral hemorrhage. PLoS One.

[80] Lu A, Tang Y, Ran R, Ardizzone TL, Wagner KR, Sharp FR (2006). Brain genomics of intracerebral hemorrhage. J Cereb Blood Flow Metab.

[81] Huang B, Krafft PR, Ma Q, Rolland WB, Caner B, Lekic T (2012). Fibroblast growth factors preserve blood-brain barrier integrity through RhoA inhibition after intracerebral hemorrhage in mice. Neurobiol Dis.

[82] Baumann N, Pham-Dinh D (2001). Biology of oligodendrocyte and myelin in the mammalian central nervous system. Physiol Rev.

[83] Hill RA, Patel KD, Goncalves CM, Grutzendler J, Nishiyama A (2014). Modulation of oligodendrocyte generation during a critical temporal window after NG2 cell division. Nat Neurosci.

[84] Cellerino A, Carroll P, Thoenen H, Barde YA (1997). Reduced size of retinal ganglion cell axons and hypomyelination in mice lacking brain-derived neurotrophic factor. Mol Cell Neurosci.

[85] Vondran MW, Clinton-Luke P, Honeywell JZ, Dreyfus CF (2010). BDNF+/− mice exhibit deficits in oligodendrocyte lineage cells of the basal forebrain. Glia.

[86] Xiao J, Wong AW, Willingham MM, van den Buuse M, Kilpatrick TJ, Murray SS (2010). Brain-derived neurotrophic factor promotes central nervous system myelination via a direct effect upon oligodendrocytes. Neuro-Signals.

[87] Miyamoto N, Maki T, Shindo A, Liang AC, Maeda M, Egawa N (2015). Astrocytes promote oligodendrogenesis after white matter damage via brain-derived neurotrophic factor. J Neurosci.

[88] Ramos-Cejudo J, Gutierrez-Fernandez M, Otero-Ortega L, Rodriguez-Frutos B, Fuentes B, Vallejo-Cremades MT (2015). Brain-derived neurotrophic factor administration mediated oligodendrocyte differentiation and myelin formation in subcortical ischemic stroke. Stroke; J Cereb Circ.

[89] McKinnon RD, Piras G, Ida JA, Dubois-Dalcq M (1993). A role for TGF-beta in oligodendrocyte differentiation. J Cell Biol.

[90] Carson MJ, Behringer RR, Brinster RL, McMorris FA (1993). Insulin-like growth factor I increases brain growth and central nervous system myelination in transgenic mice. Neuron.

[91] Aguirre A, Dupree JL, Mangin JM, Gallo V (2007). A functional role for EGFR signaling in myelination and remyelination. Nat Neurosci.

[92] Gonzalez-Perez O, Alvarez-Buylla A (2011). Oligodendrogenesis in the subventricular zone and the role of epidermal growth factor. Brain Res Rev.

[93] Gudi V, Skuljec J, Yildiz O, Frichert K, Skripuletz T, Moharregh-Khiabani D (2011). Spatial and temporal profiles of growth factor expression during CNS demyelination reveal the dynamics of repair priming. PLoS One.

[94] Olah M, Amor S, Brouwer N, Vinet J, Eggen B, Biber K (2012). Identification of a microglia phenotype supportive of remyelination. Glia.

[95] Miron VE, Boyd A, Zhao JW, Yuen TJ, Ruckh JM, Shadrach JL (2013). M2 microglia and macrophages drive oligodendrocyte differentiation during CNS remyelination. Nat Neurosci.

[96] Peferoen L, Kipp M, van der Valk P, van Noort JM, Amor S (2014). Oligodendrocyte-microglia cross-talk in the central nervous system. Immunology.

[97] Carmichael ST, Vespa PM, Saver JL, Coppola G, Geschwind DH, Starkman S (2008). Genomic profiles of damage and protection in human intracerebral hemorrhage. J Cereb Blood Flow Metab.

[98] Yang D, Han Y, Zhang J, Chopp M, Seyfried DM (2012). Statins enhance expression of growth factors and activate the PI3K/Akt-mediated signaling pathway after experimental intracerebral hemorrhage. World J Neurosci.

[99] Andres RH, Guzman R, Ducray AD, Mordasini P, Gera A, Barth A (2008). Cell replacement therapy for intracerebral hemorrhage. Neurosurg Focus.

[100] Cordeiro MF, Horn AP (2015). Stem cell therapy in intracerebral hemorrhage rat model. World J Stem Cells.

[101] Zhang H, Huang Z, Xu Y, Zhang S (2006). Differentiation and neurological benefit of the mesenchymal stem cells transplanted into the rat brain following intracerebral hemorrhage. Neurol Res.

